# The Effect of Intra-articular Injection of Hyaluronic Acid in Frozen Shoulder: a Systematic Review and Meta-analysis of Randomized Controlled Trials

**DOI:** 10.1186/s13018-022-03017-4

**Published:** 2022-03-03

**Authors:** BeiNi Mao, Run Peng, Zhong Zhang, KaiBo Zhang, Jian Li, WeiLi Fu

**Affiliations:** 1 Department of Orthopaedics Surgery, West China Hospital, Sichuan University, No. 37 Guo Xue Road, Chengdu, Sichuan 610041 People’s Republic of China; 2 Department of Orthopaedics Surgery, NO. 3 Hospital of Chengdu, Chengdu, People’s Republic of China

**Keywords:** Frozen shoulder, Hyaluronic acid, Adhesive capsulitis, Meta-analysis

## Abstract

**Background:**

Frozen shoulder (FS) is a common progressive disorder that causes restricted motion and refractory pain undermining quality of life. Intra-articular hyaluronic acid (HA) injection is a widely adopted conservative therapy relieving symptomatic FS, whereas the effect of which were contradictory and unclear in current literatures. The aim of the present study is to investigate whether intra-articular HA administration facilitates symptomatic pain relief and functional improvements in patients diagnosed with shoulder FS.

**Methods:**

The PubMed, Embase, Cochrane Library electronic databases and Google scholar were searched, from inception to 15th Jan 2022. Randomized controlled trials (RCTs) comparing intra-articular HA administration with any other non-surgical treatment in patients with FS were included. Risk of bias was evaluated using the Cochrane risk-of-bias tool and meta-analyses were undertaken to pool the data of visual analog scale for pain, range of motion (ROM) in external rotation, abduction, and flexion, as well as Shoulder Pain and Disability Index (SPADI), Constant score and American Shoulder and Elbow Surgeons (ASES).

**Results:**

The present study included 7 RCTs involving 504 patients. The results provided no support for superior pain control in patients undergoing HA injection compared with any other treatment (*p* = 0.75). Furthermore, HA group failed to exert superior improvements to other treatments in ROM concerning abduction (*p* = 0.69) and flexion (*p* = 0.33). However, HA injection was observed to facilitate functional recovery in external rotation (*p* = 0.003). In addition, the pooled data showed a significant higher SPADI score in control group than in HA group (*p* = 0.01), while no statistical significance between two groups was observed in Constant score (*p* = 0.36) and ASES (*p* = 0.76).

**Conclusions:**

The current meta-analysis suggested that HA is a beneficial treatment procedure in improving the ROM of the shoulder for patients with FS, whereas the effect in relieving pain may be equal to the existing therapy. In conclusion, Intra-articular HA injection is recommended for FS patients.

## Background

Frozen shoulder (FS), also known as adhesive capsulitis of shoulder or stiff shoulder, has been one of the most common disorders in sports medicine, which involves approximately 2–5% overall population, while the female and the elderly have been confirmed to be more susceptible [1, 2]. It is estimated that over 14,180 cases need surgical intervention per year in England, which increases healthcare cost and brings disease burden [3]. FS presents as a progressive disorder restricting both active and passive range of motion (ROM) of shoulder, nearly in all directions with more impairments in abduction and external rotation [4]. Besides limited ROM, affected patients also suffers from severe pain of the shoulder, especially at night [5]. The pathogenesis of FS remains to be defined. Idiopathic inflammation caused by inflammatory cytokines like transforming growth factor beta (TGF-β) and tumor necrosis factor alpha (TNF-α), and collagen produced by myofibroblasts, which are differentiated from fibroblasts, are thought to attribute to the contracture of the shoulder capsule. This is the most popular theory explaining the pathogenesis of FS now [6]. Although FS is considered as a self-limiting disease, the 1–3 years’ duration is too long for patients to tolerate [7]. Thus, intervention is necessary. Obviously, the principle of treatment is to relive pain and restore the ROM of the shoulder joint. A previous meta-analysis has recommended conservative treatment over surgery for lower failure rate [8]. However, a survey in British Elbow and Shoulder Society has showed that management of FS varies and is highly based on surgeons’ personal experience rather than evidence and has highlighted the need for high quality evidence [9]. There have been widely applied non-surgical treatments included steroid injection, oral therapy (particularly NSAID), physiotherapy and so on [10], while hyaluronic acid (HA) intra-articular injection has been newly proposed in recent years.

HA is a component of the synovial fluid. It has been widely applied for the treatment of knee osteoarthritis (OA), and the efficiency has been supported by plenty of researches [11, 12]. Owing to its anti-inflammation impact, the indication has been expanded to the management of FS in recent years. However, varies studies providing controversial evidences may not be sufficient to support the extensive use of HA injection [13–15].

Network meta-analysis to identified the best treatment method of FS has been conducted, and reached a conclusion that intra-articular corticosteroid injection may be the optimal management compared with other interventions. However, the study mainly focused on intra-articular corticosteroid injection, physical therapy, subacromial corticosteroid injection and arthrographic distension, while the effect of intra-articular HA injection has not been evaluated [16]. To the best of our knowledge, the effect of intra-articular HA injection on FS has not yet been studied through meta-analysis, though there have been some randomized controlled trials (RCTs). Therefore, it is still under dispute that to what degree intra-articular HA administration can help patients with FS and in what domain it can help. Hence, the objective of the present study is to investigate whether intra-articular HA administration facilitates pain relief and functional recovery in patients with FS.

## Methods

This systemic review was conducted following the instructions of the Cochrane Handbook for Systematic Reviews and reported in accordance with the Preferred Reporting Items for Systematic Review and Meta-Analyses (PRISMA) statement [17, 18].

A search of PubMed, Embase, Cochrane Library and Google scholar from the earliest record to 15th Jan 2022 was conducted. Search terms included viscosupplementation, sodium hyaluronate, hyaluronan, hyaluronic acid, scapulohumeral periarthritis, bursitis, adhesive capsulitis, periarthritis of shoulder, adhesions of the glenohumeral joint, glenohumeral arthritis, frozen shoulder, shoulder arthritis and shoulder stiffness. The search results were imported to the Endnote software by one of the authors, as well as the duplicates were removed. Two authors independently scanned the titles and the abstracts, to remove the articles that are obviously contrary to our eligibility criteria. Then the possibly included studies were read for full text. When the views of these two authors differ, the senior author was responsible to make the decision.

The following criteria were implemented to identify eligible studies: (1) Patients were diagnosed as FS, it can be also described as frozen shoulder or stiff shoulder; (2) RCTs comparing intra-articular HA injection with other non-surgical treatments; (3) outcome measurements confined to pain relief, improvement of ROM and function recovery. The exclusion criteria were: (1) animal, cadaveric or non-RCT studies; (2) studies lacking sufficient information to calculate the baseline data; (3) FS patients complicated with other shoulder diseases such as rotator cuff injury or calcific tendinitis; (4) low-quality conference summary without detailed methodological description.

Data extraction including author, publish year, group set, participants number, affected side distribution, outcome measurement, follow-up times, and demographic characteristics was conducted by 2 authors and proofread by a third author. Risk of bias was evaluated using the Cochrane risk-of-bias tool by 2 authors, and disagreements were resolved by discussion until consensus [19]. Meta-analyses were undertaken using RevMan V.5.3. The preliminary outcomes were shoulder pain, ROM and shoulder function scales. Pain assessment was calculated using visual analog scale (VAS), ROM was evaluated using the passive motion data of external rotation, flexion and abduction. Shoulder Pain and Disability Index (SPADI), Constant score and American Shoulder and Elbow Surgeons (ASES) were calculated for functional assessments. The changes between pre- and post-intervention were extracted or calculated to conducted the analysis, according to the method in the Cochrane handbook [17]. The mean difference (MD) and standard deviation (SD) were used to pool the results of different trials, using an inverse variance method. Sensitivity analysis was performed by omitting each study to see the influence to the pool results. And publication bias was evaluated by Egger test and presented as funnel plot. Statistical heterogeneity was tested by I^2^ test, while I^2^ statistic of 0–40%, 30–60%, 50–90% and 75–100% were considered to represent low, moderate, substantial, and high heterogeneity, respectively. When I^2^ < 40%, we used a fixed effects model to pool the results, otherwise, a random model was used. For all test, *p* < 0.05 represented statistically significant.

## Results

### Searching

**Fig. 1:**
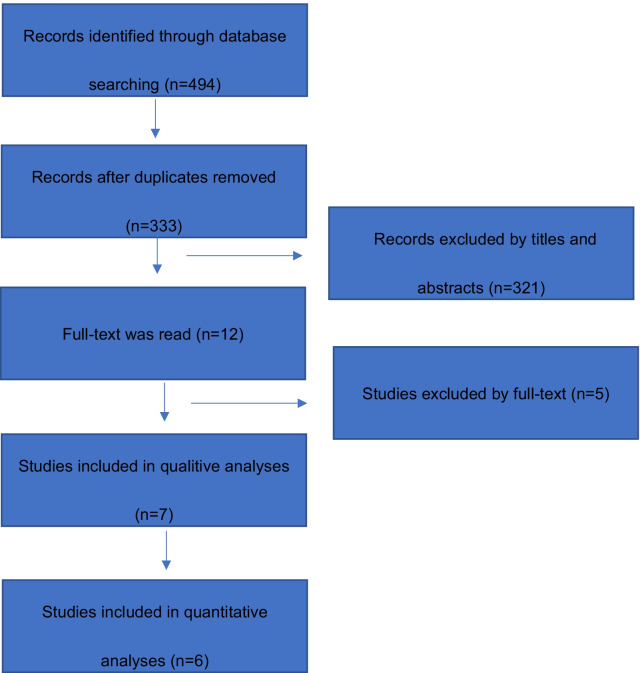
The flow chart of searching

The literature search yielded 494 studies, of which 333 were left after our removing duplicates. Next, 321 articles were excluded due to reviews, cadaveric studies, conference oral or no relevance, leaving 12 studies available for full-text. After further, full-view screening, 5 articles were excluded with reasons. Kim’s study [20] was excluded because it compared the effect of HA alone to HA with additional tramadol. Blaine’s study [21] enrolling mixed diagnosis and was not qualified for analysis. In addition, Tamai’s non- RCT study [22], as well as Yadav’s and Fabbro’s conference oral [23, 24] were also excluded. was also excluded, leaving seven studies eligible for further meta-analysis. The flow chart of searching was shown in Fig. 1.

### Study characteristics

Studies involving 504 patients dated from 1998 to 2021 [15, 25–30]. Right shoulder was more involved than left and women were susceptible. The longest follow-up period ranged from 3 to 6 months, except Akhtar’s study, [25] whose follow-up time was 4 weeks. Besides, Akhtar’s study used different scales from other studies, which was not meta- analyzed. Akhtar et al. [25], Lim et al. [26], Oh et al. [30] and Calis et al. [29] compared HA intra-articular injection with other treatments, while Park et al. [27], Hsieh et al. [28] and Rovetta and Monteforte [15] used HA as an adjunctive therapy. The characteristics of the included studies are summarized in Table 1.

**Table 1:** The characteristics of the included studies

References	Group	Number	Side (right/left)	Age	Gender (male/total)	Dose	Outcome	Follow ups
Akhtar et al. [25]	HA, NSAIDS	80, 80	92/68	45.37 ± 5.743, 37.87 ± 1.027	74/160	40 mg, once	UCLA	4 weeks
Lim et al. [26]	HA, corticosteroid	34, 34	36/30	53.8 (range 37–77 years)	19/68	20 mg, 3 times	VAS, ASES, Constant, ROM	2, 12 weeks
Park et al. [27]	HA + distension, corticosteroid	45, 45	64/26	56.33 ± 5.92, 55.23 ± 4.69	22/90	20 mg, 3 times	SPADI, VNS, ROM	2, 6, 12 weeks
Hsieh et al. [28]	HA + PT,PT	32, 31		52.6 ± 6.3, 56.4 ± 9	20/63	20 mg, 3 times	SPADI, ROM, SDQ, SF36	1.5, 3 months
Calis et al. [29]	HA, triamsinolone, PT, Stretching	10, 9, 8, 6	52/46	59.7 ± 9.81, 56.36 ± 11.3, 52.33 ± 10.1, 59.25 ± 6.8	33/90	30 mg, 2 times	VAS, ROM, Constant	15 days, 3 months
Rovetta and Montefote [15]	HA + steroid, PT + steroid	16, 14	18/14	65.8 ± 9.1, 62.3 ± 13	9/30	20 mg, 9 times	ROM,VAS	6 months
Oh et al. [30]	CS, HA, CS + HA, Saline	15, 15, 15, 15		52.3 ± 8.5, 54.5 ± 5.1, 53.5 ± 7.5, 49.4 ± 4.9	21/60	2 mL, once	ROM, VAS, UCLA, ASES, Constant, SPADI	1 week and 1, 3, 6 months

HA, hyaluronic acid; NSAIDS, non-steroidal anti-inflammatory drugs; UCLA, University of California Los-Angeles; VAS, visual analog scale; ASES, American Shoulder and Elbow Surgeon; ROM, range of motion; SPADI, Shoulder Pain and Disability Index; VNS, Verbal Numeric Scale; PT, physical therapy; SDQ, Shoulder Disability Questionnaire; SF36, Medical Outcomes Study 36-Item Short-Form Health Survey

### Risk of bias

**Fig. 2:**
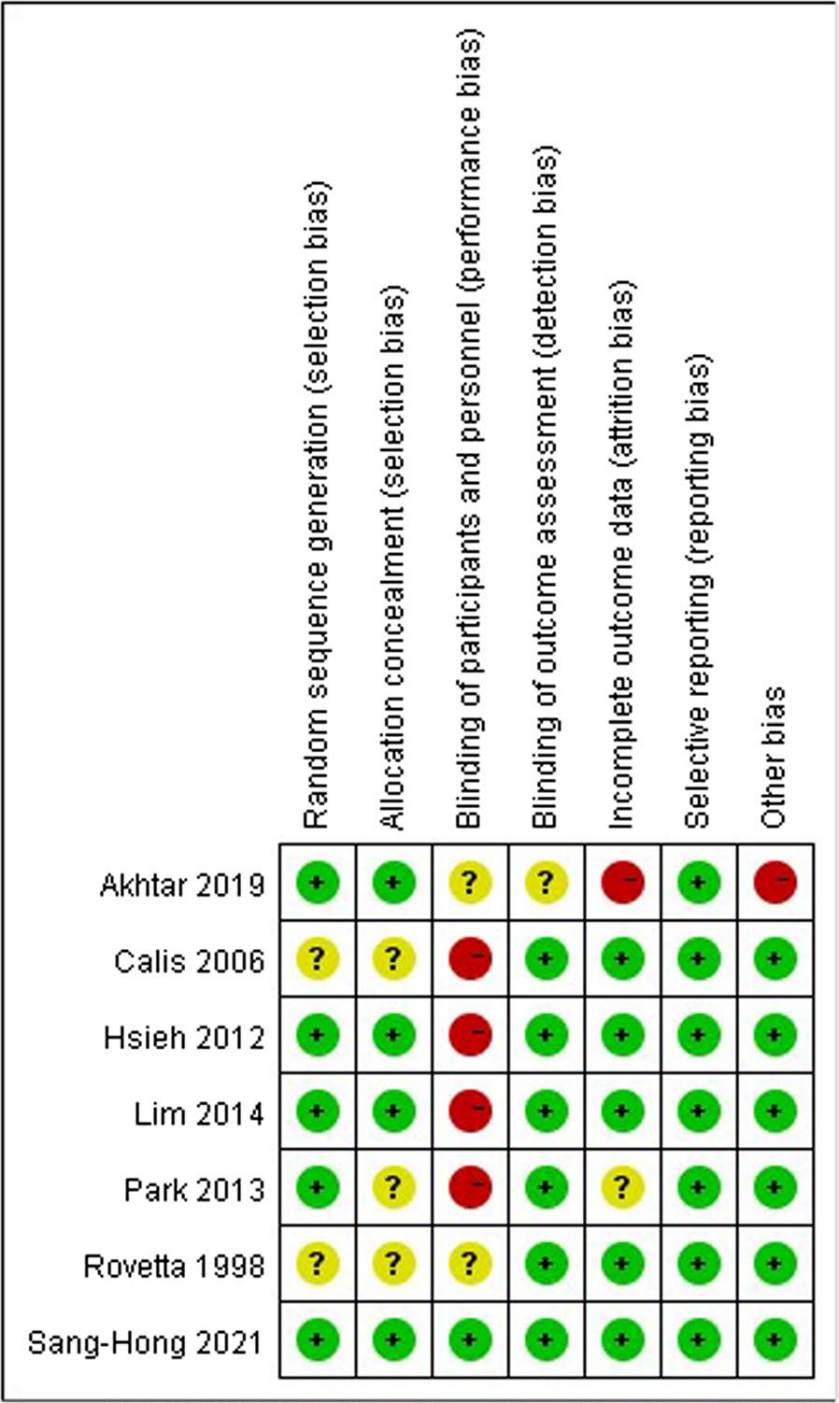
The risk of bias assessment

Five studies [25–29] had a high risk of performance bias, mainly because the different intervention failed to achieve blind requirements. Two study [15, 29] did not describe random procedure clearly. Three studies [15, 27, 29] did not describe allocation clearly. Two studies [15, 25] did not describe the blinding of participates and personnel, and we cannot tell from their trails, either. The blinding of outcome assessment and attrition bias were not clear in one study, respectively [25, 27]. The result of risk of bias assessment was shown in Fig. 2.

### Pain relief

**Fig. 3:**
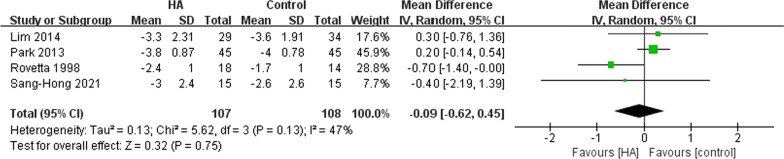
The pooled data of VAS

All studies involved pain relief while two [15, 26] used VAS to measure the outcomes. Besides, Park et al. [27] used Verbal Numeric Scale (VNS) to assess pain, which was equal to VAS according to the author’s description. Finally, the remaining 4 studies were included for meta-analysis. The results (*p* = 0.75) showed that HA did not yield a better efficacy in pain relief compared with other therapies. The result was shown in Fig. 3.

### Improvement of ROM

**Fig. 4:**
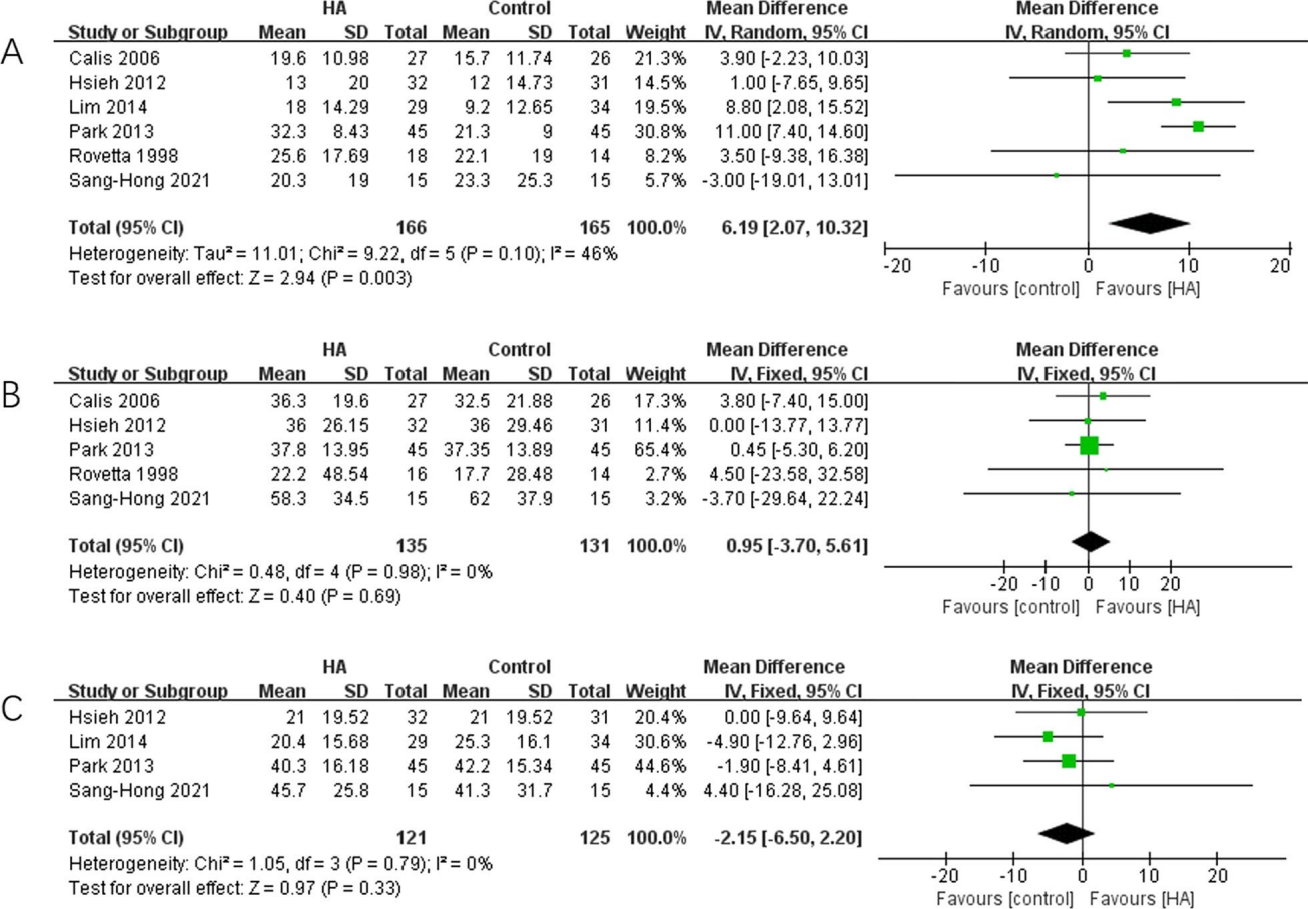
The pooled data of improvements of ROM. **A** External rotation; **B** abduction; **C** flexion

Six studies [15, 26–30] presented the shoulder ROM changes including external rotation, abduction and flexion, which were meta-analyzed. The results showed better improvements in HA group in external rotation (*p* = 0.003), but not in abduction (*p* = 0.69) and flexion (*p* = 0.33), compared with control group. The results were shown in Fig. 4.

### Functional scales

**Fig. 5:**
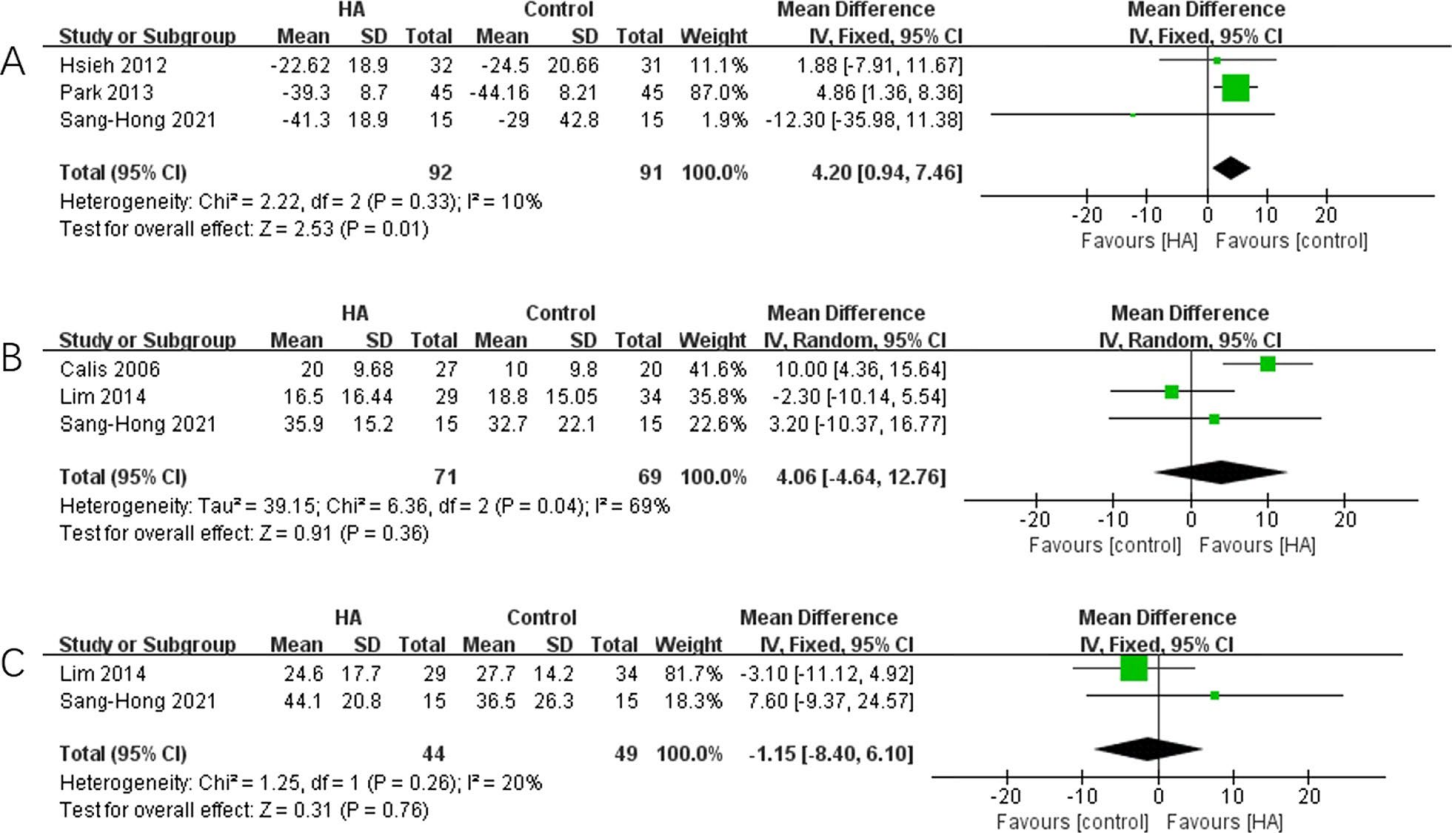
The pooled data of functional scales. **A** SPADI; **B** constant score; **C** ASES

Three studies [27, 28, 30] evaluated the SPADI and the pooled data showed control group had a better improvement than HA group (*p* = 0.01), with a low heterogeneity (*I*^2^ = 10%). Three studies [26, 29] evaluated the Constant score and the pooled data showed no difference between HA and control group (*p* = 0.36). While ASES showed no difference in HA and control group, either (*p* = 0.76). The results of the functional scales were shown in Fig. 5.

### Sensitive analysis

The sensitive analysis showed no study obviously influenced the results.

### Publication bias

**Fig. 6:**
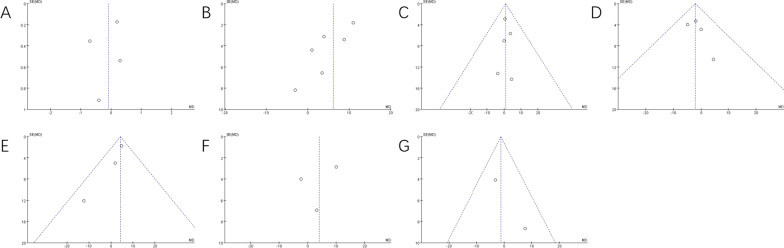
funnel plot in **A** VAS; **B** external rotation; **C** abduction; **D** flexion; **E** SPADI; **F** constant score; **G** ASES

There was a slight publication bias according to the funnel plot (Fig. 6).

## Discussion

FS is associated with continuous shoulder pain and loss of ROM, and can lead to shoulder disability and disrupted sleep [31]. Despite the current retrospective study [32] considering FS as a self-limited disease, treatments aimed at relieving pain and restoring shoulder motion and function was recommended [33] with favorable choice remained. In addition to nonsteroidal anti-inflammatory drugs, intra-articular corticosteroid administration and physical therapy, intra-articular injection of HA is gaining growing popularity in comparison with other conventional therapies. Some studies have compared different treatments for FS, whereas none involved intra-articular injection of HA [16, 34]. Hence, the current study aimed to quantitatively evaluate the effect of intra-articular HA injection on FS patients concerning pain relief and functional improvements.

Our results showed favorable outcomes in intra-articular HA injection, but failed to exert superior effect compared to other conventional therapy in pain relief. Since FS is a self-limited disease involving 3 phases. A constant pain appears early in painful freezing phase, and gradually subsides in adhesive phase. And the reduction of glenohumeral movements starts to improve until resolution phase. the between-approaches comparisons of pain-relieving effect might be complicated to make when FS turns into adhesive phase, in which the pain naturally relieves [2]. According to the assessing timepoints, pain might decrease spontaneously in both experimental and control groups leading to no statistical significance between groups. In addition, combination of treatments in controls may also affect the analysis. Lim et al. [26] and Park et al. [27] used intra-articular injection of corticosteroid as control, while Rovetta and Monteforte [15] used HA as an adjunctive therapy to corticosteroid plus physical therapy. Since corticosteroid is characterized with strong analgesic effect, studies enrolling usage of corticosteroid may suppress symptomatic pain manifestation [16, 34]. Some studies proved that corticosteroid contributed to anti-inflammation earlier than HA [35]. The pain-relieving effect of HA might be prominent in long-term control, which was supported by some studies [21, 36, 37]. Akhtar et al. confirmed the effect of HA in pain relief by UCLA pain scale [25] by conducting a RCT. An animal experiments also suggested that HA could reduce the concentration of inflammatory mediators such as prostaglandins, fibronectin, and cyclic adenosine monophosphate [38]. According to our results, HA has a comparable effect of pain relief with corticosteroid injection, though not superior. Given the side-effects of corticosteroid resulting in periarticular calcification, cutaneous atrophy, cutaneous depigmentation, tendon rupture and avascular necrosis [39–41], we suggested that intra-articular injection of HA is a favorable option in pain relief for FS patients.

The results of functional assessments, as shown in Fig. 4, clearly imply that the patients with HA interventions tend to have higher ROM values in external rotation than the control. When evaluating the remaining ROM aspects, we did not find statistical significances concerning ROM in abduction and flexion. Given the mixed results on the efficacy of HA in functional improvements, its application in patients with restricted ROM should be discussed. The ROM calculations, varied in individual studies, that Cails et al. [29] and Park et al. [27] assessed passive ROM and Hsieh et al. [28] and Oh et al. [30] assessed both active and passive ROM. Lim et al. [26] and Rovetta and Monteforte [15] haven’t mentioned it but we can judge from their context that they also used passive ROM, too. Therefore, the passive ROM data were calculated to perform data synthesis. The follow-up time was 3 months for most studies [26, 28, 29], while Rovetta and Monteforte [15] assessed at 6 weeks and Park et al. [27] and Oh et al. [30] assessed at 6 months, so the follow up time were all less than 6 months. Except for Hsieh’s, all studies used corticosteroid in controls. may also affect the performances of HA as discussed above [15, 26, 27, 29]. Overall, the eligible individual studies in the current meta-analysis presented low heterogeneity, which may suggest the credibility of the results. All individual study included for quantitative analysis reported improved ROM in external rotation, and eventually achieved a result favor for HA. From the perspective of directions of ROM, external rotation was well-established as the most important and sensitive direction in the motion of shoulder [4, 29], which also frequently selected as the representative of movements of shoulder [5, 42]. The superior improvement in external rotation suggested HA have a superior effect in functional recovery. The reason why significance was not found in abduction and flexion may be the limited sample size or a low sensitivity of these two directions. In addition, corticosteroid usage in control groups may also affect the comparison results. Therefore, the higher external rotation ROM values led us to considered HA injection as the better qualified treatment in improving ROM of shoulder in FS patients, compared to the existing therapy.

As for functional assessments, results of Constant and ASES showed no statistical significance between HA and control group. Interestingly, HA group showed worse outcomes than control group in SPADI, which is a self-rating scale consisting of pain scale and disability scale [43]. The result in SPADI was pooled from three studies [27, 28, 30], while Park’s study weighted 87%. Among them, two studies used HA as an adjunctive therapy to the various treatments. In Park’s study, they used 0.5% lidocaine (18 mL) for capsular distension before HA administration in HA group, while 0.5% lidocaine (4 mL) plus triamcinolone (40 mg/mL; 1 mL) in control group without capsular distension. As reported, twelve patients in treatment group suffered pain during capsular distension, which may influence the functional exercise, thus affect the SPADI [27]. Since capsular distension is the main reason for the poor results in SPADI, the results may not be able to illustrate that the effect of HA injection was inferior to other treatments. After comprehensively analyzing the results of Constant and ASES score, we tend to conclude that HA injection may possess a similar effect than the existing treatments.

Some previous reviews [44, 45] have discussed the effect of HA on FS. Papalia et al. [44] stated that HA is effective, but not as effective as other conventional treatments. The study included two non-RCTs, which decreased its level of evidence. Lee et al. [45] included 4 trails and conducted a systematic review to compare the effect of HA and other conventional therapies on FS patients, which suggested that HA yielded limited effect, whether adopted individually or in combination. However, the absence of quantitative analysis in this study due to data limitation may further undermine its credibility. Comparing with the previous review, the current study enlarged samples with two recently published RCTs, and meta-analysis may contribute to more reliable results. Harris et al. [46] conducted a systematic review and concluded that HA injection into the glenohumeral joint significantly improved shoulder ROM, constant scores, and pain at short-term follow-up following treatment of FS while isolated intra-articular HA injection presented significantly better outcomes than control. Though we reached a similar conclusion, his study included too many low-level evidence studies, which made his conclusion less convincing, as well as meta-analysis was not performed by them. Our study confirmed his view with a more typical inclusion and exclusion criteria, and meta-analysis made the conclusion more reliable.

Overall, the present meta-analysis suggested that HA intra-articular injection displayed non-inferior effect in pain-relieving and superior performance in functional improvements compared with other conventional treatment. Some meta-analyses have investigated the optimal treatments of FS [16, 34], of which Challoumas’s is the latest well-designed study with largest samples. This net-work meta-analysis recommended corticosteroid intra-articular injection for FS patients within 1-year duration. Challoumas et al. argued for its earlier benefits in contrast to interventions with detailed comparisons regarding physiotherapy, intra-articular corticosteroid, subacromial corticosteroid, arthrographic distension plus intra-articular corticosteroid and no treatment or placebo [16]. However, the major concern is the absence of HA injection comparison. To the best of our knowledge, the current study is the first meta-analysis with the largest available samples investigating the effect on FS between HA injection and any other conservative interventions.

The HA molecule has properties of both viscous and elastic materials, and this property suggests that it may have lubricate effect, as well as anti-adhesion effect [47]. The adhesion of FS comes from the deposition of type I and type III collagen by fibroblasts and myofibroblasts. It is also widely believed to be caused by a synovial inflammation [2]. HA as a lubricant, directly increases the viscoelasticity of the joint, and promotes the release of adhesions. And HA improves synovial fluid concentrations and changes synovium abnormalities, which also reduces friction [48]. Besides, its effect in anti-inflammatory actions and protection of cartilage may also be helpful [11]. In summary, it is a result of comprehensive action that HA helps shoulder return to normal and promotes the release of adhesions.

There were several limitations in this meta-analysis. First, our meta-analysis was conducted without classifications concerning individual stages of FS, which may add to the confounding factors not stratified in the current analysis. However, we have attempted to search eligible studies, whereas the eligible studies did not recruit patients in accordance with specific criteria, and the participates varies in stages. Second, we failed to make stratified subgroup analyses to calculate separate conventional treatment comparisons. However, we have endeavored to calculated as much original data as much as possible, which was limited due to data absence. Third, potential bias may exist in the current study due to lack of ethnical issues, language of publications, and minor publication bias, which could be resolved by further meta-analysis based on larger samples included. Although these limitations exist, we believe that this high-level of evidence research is helpful for clinical decision-making.

## Conclusions

HA intra-articular injection is a beneficial treatment procedure in improving the ROM of the shoulder for patients with FS, and the effect in relieving pain is equal to existing therapies. In conclusion, intra-articular HA injection is recommended for FS patients.

## Data Availability

All data generated or analyzed during this study are included in this article. The data are available from the corresponding author upon reasonable request.

## Abbreviations

FS: Frozen Shoulder
HA: Hyaluronic acid
RCT: Randomized controlled trial
VAS: Visual analog scale
ROM: Range of motion
SPADI: Shoulder Pain and Disability Index
TGF-β: Transforming growth factor beta
TNF-α: Tumor necrosis factor alpha
OA: Osteoarthritis
PRISMA: Preferred Reporting Items for Systematic Review and Meta-Analyses
MD: Mean difference
SD: Standard deviation

## References

[1] Neviaser RJ, Neviaser TJ (1987). The frozen shoulder. Diagnosis and management. Clin Orthop Relat Res.

[2] Dias R, Cutts S, Massoud S (2005). Frozen shoulder. BMJ.

[3] Kwaees TA, Charalambous CP (2015). Rates of surgery for frozen shoulder: an experience in England. Muscles Ligaments Tendons J.

[4] Redler LH, Dennis ER (2019). Treatment of adhesive capsulitis of the shoulder. J Am Acad Orthop Surg.

[5] Reeves B (1975). The natural history of the frozen shoulder syndrome. Scand J Rheumatol.

[6] Georgiannos D, Markopoulos G, Devetzi E, Bisbinas I (2017). Adhesive capsulitis of the shoulder. Is there consensus regarding the treatment? A Comprehensive Review. Open Orthop J..

[7] Ewald A (2011). Adhesive capsulitis: a review. Am Fam Physician.

[8] Longo UG, Ciuffreda M, Locher J, Buchmann S, Maffulli N, Denaro V (2018). The effectiveness of conservative and surgical treatment for shoulder stiffness: a systematic review of current literature. Br Med Bull.

[9] Kwaees TA, Charalambous CP (2014). Surgical and non-surgical treatment of frozen shoulder. Survey on surgeons treatment preferences. Muscles Ligaments Tendons J.

[10] Cavalleri E, Servadio A, Berardi A, Tofani M, Galeoto G (2020). The effectiveness of physiotherapy in idiopathic or primary frozen shoulder: a systematic review and meta-analysis. Muscles Ligaments Tendons J.

[11] Iwata H (1993). Pharmacologic and clinical aspects of intraarticular injection of hyaluronate. Clin Orthop Relat Res.

[12] Lussier A, Cividino AA, McFarlane CA, Olszynski WP, Potashner WJ, De Médicis R (1996). Viscosupplementation with hylan for the treatment of osteoarthritis: findings from clinical practice in Canada. J Rheumatol.

[13] Itokazu M, Matsunaga T (1995). Clinical evaluation of high-molecular-weight sodium hyaluronate for the treatment of patients with periarthritis of the shoulder. Clin Ther.

[14] Leardini G, Perbellini A, Franceschini M, Mattara L (1988). Intra-articular injections of hyaluronic acid in the treatment of painful shoulder. Clin Ther.

[15] Rovetta G, Monteforte P (1998). Intraarticular injection of sodium hyaluronate plus steroid versus steroid in adhesive capsulitis of the shoulder. Int J Tissue React.

[16] Challoumas D, Biddle M, McLean M, Millar NL (2020). Comparison of treatments for frozen shoulder: a systematic review and meta-analysis. JAMA Netw Open.

[17] Higgins JPTGS. Cochrane handbook for systematic reviews of interventions (Version 5.1.0) 2011.

[18] Moher D, Liberati A, Tetzlaff J, Altman DG (2009). Preferred reporting items for systematic reviews and meta-analyses: the PRISMA statement. BMJ.

[19] Higgins JP, Altman DG, Gøtzsche PC, Jüni P, Moher D, Oxman AD (2011). The Cochrane Collaboration's tool for assessing risk of bias in randomised trials. BMJ.

[20] Kim KH, Suh JW, Oh KY (2017). The effect of intra-articular hyaluronate and tramadol injection on patients with adhesive capsulitis of the shoulder. J Back Musculoskelet Rehabil.

[21] Blaine T, Moskowitz R, Udell J, Skyhar M, Levin R, Friedlander J (2008). Treatment of persistent shoulder pain with sodium hyaluronate: a randomized, controlled trial. A multicenter study. J Bone Joint Surg Am.

[22] Tamai K, Yamato M, Hamada J, Mashitori H, Saotome K (1999). Response of frozen shoulder to intraarticular corticosteroid and hyaluronate: a quantitative assessment with dynamic magnetic resonance imaging. Dokkyo J Med Sci.

[23] Yadav SL (2016). Role of intra-articular hyaluronic acid in management of periarthritis shoulder. Pain Pract.

[24] Fabbro E, Lacelli F, Ferrero G, Orlandi D, Sconfienza LM, Serafini G (2012). Ultrasound-guided treatment of adhesive capsulitis (frozen shoulder) with intraarticular injection of mepivacaine and steroid VS hyaluronic acid (HA). Skeletal Radiol.

[25] Akhtar M, Nadeem RDA, Shah Gillani SF, Cheema OI, Nadeem MR (2019). Comparison of intra articular NSAID (ketorolac) injection versus hyaluronic acid injection for the mean decrease of pain score (according to UCLA shoulder rating scale) in the management of adhesive capsulitis. Pak J Pharm Sci.

[26] Lim TK, Koh KH, Shon MS, Lee SW, Park YE, Yoo JC (2014). Intra-articular injection of hyaluronate versus corticosteroid in adhesive capsulitis. Orthopedics.

[27] Park KD, Nam HS, Lee JK, Kim YJ, Park Y (2013). Treatment effects of ultrasound-guided capsular distension with hyaluronic acid in adhesive capsulitis of the shoulder. Arch Phys Med Rehabil.

[28] Hsieh LF, Hsu WC, Lin YJ, Chang HL, Chen CC, Huang V (2012). Addition of intra-articular hyaluronate injection to physical therapy program produces no extra benefits in patients with adhesive capsulitis of the shoulder: a randomized controlled trial. Arch Phys Med Rehabil.

[29] Calis M, Demir H, Ulker S, Kirnap M, Duygulu F, Calis HT (2006). Is intraarticular sodium hyaluronate injection an alternative treatment in patients with adhesive capsulitis?. Rheumatol Int.

[30] Oh SH, Sung WS, Oh SH, Jo CH (2021). Comparative analysis of intra-articular injection of steroid and/or sodium hyaluronate in adhesive capsulitis: prospective, double-blind, randomized, placebo-controlled study. JSES Int.

[31] Jones S, Hanchard N, Hamilton S, Rangan A (2013). A qualitative study of patients' perceptions and priorities when living with primary frozen shoulder. BMJ Open.

[32] Diercks RL, Stevens M (2004). Gentle thawing of the frozen shoulder: a prospective study of supervised neglect versus intensive physical therapy in seventy-seven patients with frozen shoulder syndrome followed up for two years. J Shoulder Elbow Surg.

[33] Wong CK, Levine WN, Deo K, Kesting RS, Mercer EA, Schram GA (2017). Natural history of frozen shoulder: fact or fiction? A systematic review. Physiotherapy.

[34] Lin MT, Hsiao MY, Tu YK, Wang TG (2018). Comparative efficacy of intra-articular steroid injection and distension in patients with frozen shoulder: a systematic review and network meta-analysis. Arch Phys Med Rehabil.

[35] Bellamy N, Campbell J, Robinson V, Gee T, Bourne R, Wells G (2006). Intraarticular corticosteroid for treatment of osteoarthritis of the knee. Cochrane Database Syst Rev.

[36] Abate M, Pulcini D, Di Iorio A, Schiavone C (2010). Viscosupplementation with intra-articular hyaluronic acid for treatment of osteoarthritis in the elderly. Curr Pharm Des.

[37] Brander VA, Gomberawalla A, Chambers M, Bowen M, Nuber G (2010). Efficacy and safety of hylan G-F 20 for symptomatic glenohumeral osteoarthritis: a prospective, pilot study. PM R.

[38] Conduah AH, Baker CL, Baker CL (2009). Managing joint pain in osteoarthritis: safety and efficacy of hylan G-F 20. J Pain Res.

[39] Habib GS, Saliba W, Nashashibi M (2010). Local effects of intra-articular corticosteroids. Clin Rheumatol.

[40] Blanco I, Krähenbühl S, Schlienger RG (2005). Corticosteroid-associated tendinopathies: an analysis of the published literature and spontaneous pharmacovigilance data. Drug Saf.

[41] Parikh JR, Houpt JB, Jacobs S, Fernandes BJ (1993). Charcot's arthropathy of the shoulder following intraarticular corticosteroid injections. J Rheumatol.

[42] Rizk TE, Pinals RS (1982). Frozen shoulder. Semin Arthritis Rheum.

[43] Roach KE, Budiman-Mak E, Songsiridej N, Lertratanakul Y (1991). Development of a shoulder pain and disability index. Arthritis Care Res.

[44] Papalia R, Tecame A, Vadala G, Russo F, Perna M, Papalia G (2017). The use of hyaluronic acid in the treatment of shoulder capsulitis: a systematic review. J Biol Regul Homeost Agents.

[45] Lee LC, Lieu FK, Lee HL, Tung TH (2015). Effectiveness of hyaluronic acid administration in treating adhesive capsulitis of the shoulder: a systematic review of randomized controlled trials. Biomed Res Int.

[46] Harris JD, Griesser MJ, Copelan A, Jones GL (2011). Treatment of adhesive capsulitis with intra-articular hyaluronate: a systematic review. Int J Shoulder Surg.

[47] Pelletier JP, Martel-Pelletier J (1993). The pathophysiology of osteoarthritis and the implication of the use of hyaluronan and hylan as therapeutic agents in viscosupplementation. J Rheumatol Suppl.

[48] Altman RD, Akermark C, Beaulieu AD, Schnitzer T (2004). Efficacy and safety of a single intra-articular injection of non-animal stabilized hyaluronic acid (NASHA) in patients with osteoarthritis of the knee. Osteoarthritis Cartilage.

